# Exploring the utility of a latent variable as comprehensive inflammatory prognostic index in critically ill patients with cerebral infarction

**DOI:** 10.3389/fneur.2024.1287895

**Published:** 2024-01-15

**Authors:** Chang Shu, Chenguang Zheng, Guobin Zhang

**Affiliations:** ^1^Tianjin Key Laboratory of Cerebral Vascular and Neurodegenerative Diseases, Tianjin Neurosurgical Institute, Tianjin Huanhu Hospital, Tianjin, China; ^2^Tianjin Key Laboratory of Brain Science and Neural Engineering, Tianjin University, Tianjin, China; ^3^Neural Intensive Care Unit, Tianjin Huanhu Hospital, Tianjin, China

**Keywords:** cerebral infarction, prognostic index, mortality, exploratory factor analysis, survival analysis

## Abstract

**Objective:**

We introduce the comprehensive inflammatory prognostic index (CIPI), a novel prognostic tool for critically ill cerebral infarction patients, designed to meet the urgent need for timely and convenient clinical decision-making in this high-risk patient group.

**Methods:**

Using exploratory factor analysis on selected indices—neutrophil to lymphocyte ratio (NLR), systemic inflammation response index (SIRI), and systemic immune inflammation index (SIII)—we derived CIPI, a latent variable capturing their combined predictive power. Data from 1,022 patients in the Medical Information Mart for Intensive Care (MIMIC)-IV database were used to develop CIPI-based survival models, with the robustness and area under the receiver operating characteristic curve (AUC) performance of CIPI validated against an independent dataset of 326 patients from the MIMIC-III CareVue subset. The CIPI’s predictive power for in-hospital and intensive care unit (ICU) mortality was assessed through Kaplan–Meier analysis, univariate and multivariate Cox regression models, and time-dependent AUC analysis. Linearity, subgroup sensitivity analyses and interaction effects with CIPI were also evaluated.

**Results:**

CIPI was an independent prognostic factor, demonstrating a statistically significant association with in-hospital and ICU mortality, when assessed as a continuous and a categorical variable. It showed a linear relationship with mortality rates and demonstrated stability across most subgroups, with no significant interactions observed. Its predictive capabilities for in-hospital and ICU mortality among critically ill cerebral infarction patients matched those of established prognostic indices in the MIMIC database.

**Conclusion:**

Our study indicates that CIPI is a reliable and effective prognostic tool for critically ill cerebral infarction patients in predicting in-hospital and ICU mortality. Its straightforward calculation, rooted in routine blood tests, enhances its practicality, promising significant utility in clinical settings.

## Introduction

Critically ill patients suffering from cerebral infarction, a distinct and severe form of ischemic stroke, constitute a subgroup with substantial healthcare needs (1). Marked by significant neurological deficits and prone to multi-system complications and comorbidities, these individuals often require intensive care unit (ICU) management (2, 3). The seriousness of their condition is mirrored in the elevated mortality rates observed during their hospital stays (4, 5), underscoring the pressing need for enhanced prognostic tools. Precisely and timely predicting which patients in the ICU with cerebral infarction are most at risk of adverse outcomes is a critical clinical imperative; it is a key determinant that can profoundly influence the direction of therapeutic strategies and patient care (6). Building on previous research, our study is committed to identifying an independent and comprehensive prognostic index that is not only effective but also accessible in predicting in-hospital and ICU mortality for this at-risk patient group.

Increasingly, prognostic significance in critically ill patients with cerebral infarction is being assigned to inflammation-related composite hematological indices (7–9). Indices such as the neutrophil to lymphocyte ratio (NLR), systemic inflammation response index (SIRI), and the systemic immune inflammation index (SIII) have all been independently associated with in-hospital mortality in critically ill patients with cerebral infarction (8–10). However, including these highly correlated composite hematological indices in survival analysis models simultaneously can lead to common issues like multicollinearity, complicating their interpretation and potentially weakening the model’s overall predictive accuracy (11–13). To overcome this issue, our study implemented a novel, data-driven approach. Initially, we performed a preliminary screening of various composite inflammatory indices to select those with the best predictive capabilities. After that NLR, SIRI, and SIII were selected based on their superior predictive abilities. Subsequently, we applied exploratory factor analysis (EFA) to these chosen indices, leading to the identification of a latent variable, which we have named the comprehensive inflammatory prognostic index (CIPI). CIPI encapsulates the predictive capacities of each of the three individual indices, enhancing their combined utility and potentially addressing the inherent limitations by integrating highly correlated and collinear variables into a single model.

The aim of our study was to identify and confirm CIPI as an independent prognostic factor for critically ill patients with cerebral infarction, with a specific focus on in-hospital and ICU mortality, and to evaluate its predictive performance. This endeavor seeks to equip clinicians with a more effective and convenient tool, thereby enhancing their decision-making capabilities in patient care and potentially improving treatment outcomes.

## Methods

### Data source

In our study, we predominantly used the Medical Information Mart for Intensive Care (MIMIC)-IV (v2.2) database, a valuable and comprehensive resource for critical care research (14, 15). To enhance the robustness of our findings, we performed an external validation using the CareVue subset of the MIMIC-III (v1.4) database (15, 16). This subset supplied an independent collection of patient data not found in MIMIC-IV, enabling us to circumvent any duplication due to overlapping admissions. The first author, after completing the National Institutes of Health’s Protecting Human Research Participants web course, secured permission to access these datasets. The research committees at the Massachusetts Institute of Technology and Beth Israel Deaconess Medical Center approved their use for research, and a waiver of informed consent was granted.

### Data extraction and variables selection

**Figure 1:**
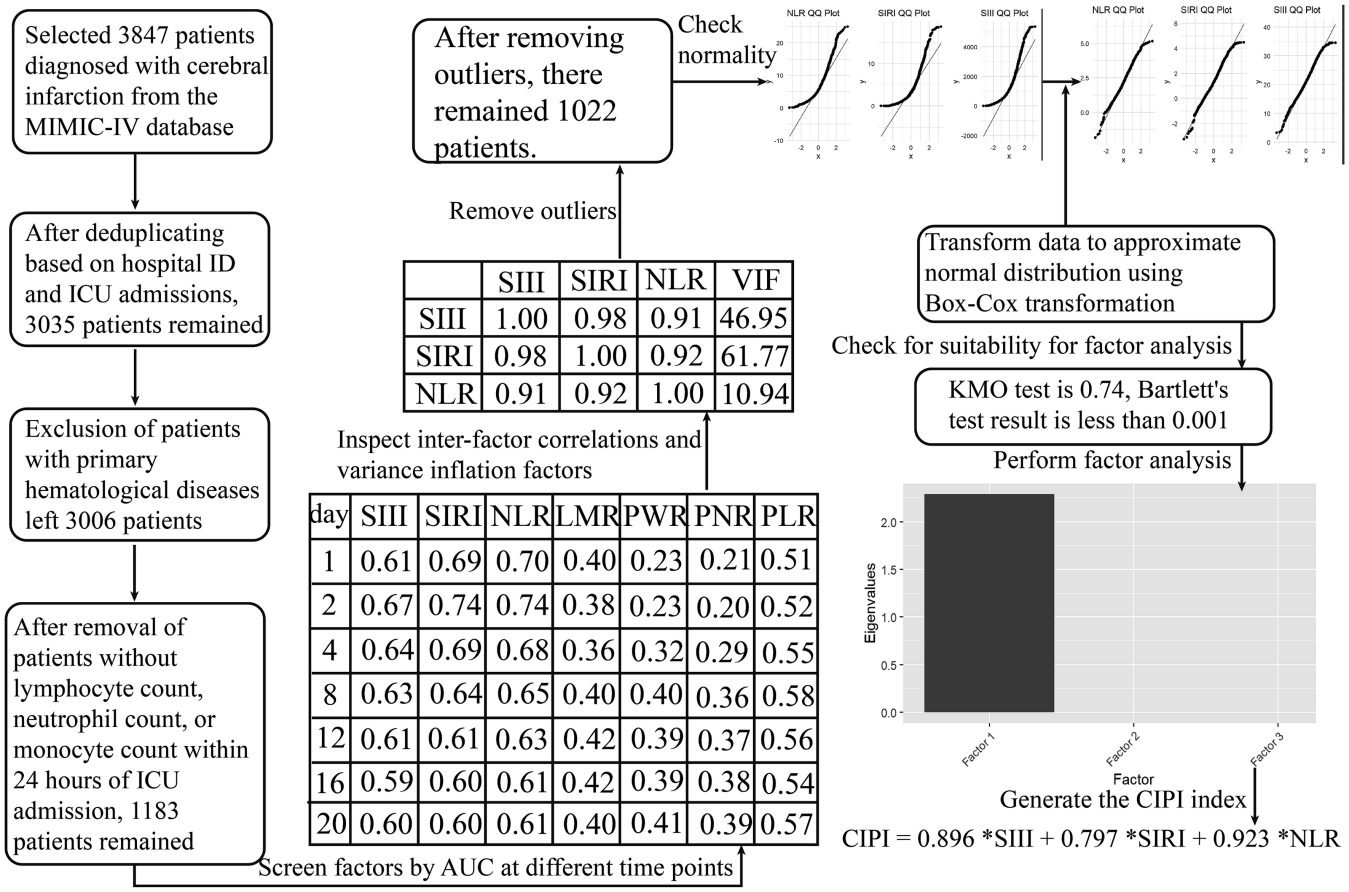
Computation process of the latent variable for survival analysis. We conducted a thorough evaluation of multiple inflammation-related composite hematological indices, using time-dependent AUC analysis as the criterion to identify suitable manifest variables for exploratory factor analysis. Ultimately, NLR, SIRI, and SIII were selected for the factor analysis. The initial inflammation-related composite hematological indices evaluated were as follows: SIRI (systemic inflammation response index) = (neutrophil * monocyte)/lymphocyte; SIII (systemic immune inflammation index) = (platelet * neutrophil)/lymphocyte; NLR (neutrophil to lymphocyte ratio) = neutrophil/ lymphocyte; LMR (lymphocyte to monocyte ratio) = lymphocyte/monocyte; PWR (platelet to white blood cell ratio) = platelet /white blood cell; PNR (platelet to neutrophil ratio) = platelet /neutrophil; PLR (platelet to lymphocyte ratio) = platelet/lymphocyte.

Data extraction was executed using Python’s SQLAlchemy and pandas libraries, connected to a PostgreSQL (version 13.7.2) database, and leveraging Structured Query Language (SQL) for the necessary queries. Critically ill patients diagnosed with cerebral infarction were identified in the database by the following International Classification of Diseases, Ninth Revision (ICD-9) codes: 43,301, 43,311, 43,321, 43,331, 43,381, 43,391, 43,401, 43,411, and 43,491; as well as the Tenth Revision (ICD-10) code I63. The deduplication process was conducted based on clinical rationale, prioritizing the initial cerebral infarction diagnosis for each unique ICU stay. In cases of multiple ICU stays within a single hospital admission, any occurrence of death was considered a single mortality event. If no death occurred, the record corresponding to the patient’s first ICU stay was selected. We eliminated all patients with primary blood diseases by using ICD codes, as well as patients for whom records of lymphocytes, monocytes, neutrophils or platelet were absent (Figure 1).

In the selection of covariates from the MIMIC-IV database, we prioritized those with no missing values or those where less than 20% of the data was missing, with missing values for relevant variables filled in using the KNNImputer method (17). Notably, initial post-admission results were used for laboratory indicators. Averages for vital signs were taken from the first 24 h in the ICU, during which time established and widely-recognized prognostic scores were also assessed; these scores were later compared with the CIPI to evaluate predictive accuracy.

For external validation of CIPI, the same extraction, deduplication, and data processing methods were applied to the MIMIC-III CareVue subset, following the exact standards used with the MIMIC-IV database. The data processing included outlier exclusion, data transformation, standardization, and latent variable weight extraction. The MIMIC-III CareVue subset was employed to validate univariate Cox regression analysis of CIPI, SIRI, SIII, and NLR, as well as the time-dependent area under the receiver operating characteristic curve (AUC) analysis for predicting in-hospital and ICU mortality using CIPI.

### Clinical outcomes

The primary outcome measure in the present study was in-hospital mortality, with ICU mortality as the secondary outcome.

### Statistical analysis

Initially, we applied time-dependent AUC analysis to the raw data from the MIMIC-IV database, aiming to identify appropriate manifest variables for EFA (Figure 1). This analysis focused on a broad spectrum of composite hematological indices. These selected manifest variables (SIRI, SIII, and NLR) underwent correlation and multicollinearity analyses, outlier removal, and normality testing via the Shapiro–Wilk test, histograms, and Q-Q plots. To enhance their normality, we applied a Box-Cox transformation. Following the standardization of these variables, they were subjected to Kaiser-Meyer-Olkin and Bartlett’s Test of Sphericity to assess their suitability for EFA. Subsequently, we conducted EFA, generating a factor scoring coefficient equation that enabled us to calculate the latent variable score and evaluate its contribution (Figure 1).

We treated CIPI both as a continuous variable and divided it into four groups based on quartiles (Q1, Q2, Q3, Q4). Furthermore, all other variables were similarly divided into quartile-based groups corresponding to the CIPI quartiles for subsequent analyses and comparisons. Continuous variables are reported as the median and interquartile range. Categorical variables are represented as frequency counts and percentages. The quartile groups were compared using the Kruskal-Wallis test for continuous variables due to their non-parametric distribution, and the Chi-square test was employed for categorical variables.

Kaplan–Meier analysis evaluated in-hospital and ICU mortality across CIPI quartiles (Q1–Q4), with survival differences tested using log-rank tests. We assessed the linearity of CIPI using a restricted cubic spline with default knots. This was followed by a comparison between linear and non-linear Cox models via the Akaike Information Criterion (AIC) and an ANOVA test. We applied Cox proportional hazards models to compute the hazard ratios (HR) and 95% confidence intervals (CI) associated with the CIPI, using both its continuous form and quartile divisions (Q1–Q4), with reference to Q1. Model covariates were chosen based on clinical relevance, prognostic value from literature, and expert opinion. Trend *p*-values across quartiles were additionally computed to assess the dose–response relationship. We conducted sensitivity analyses to further evaluate the robustness of our findings. In these analyses, CIPI’s predictive value was assessed across different patient subgroups, including factors such as sex, age, marital status, whether cerebral infarction was the first diagnosis, and the presence of comorbidities like diabetes, heart failure, kidney disease, and sepsis. Separate Cox proportional hazards models were utilized for each subgroup, and the value of p for interaction was assessed to determine the existence of a significant interaction effect. Time-dependent AUC analysis was employed to assess the predictive accuracy of the CIPI in comparison with other established ICU patient prognosis scores available in the MIMIC database. Detailed information on these scores can be found at: https://github.com/MIT-LCP/mimic-code/tree/main/mimic-iv/concepts_postgres/score. The Delong method was used to ascertain the statistical significance of AUC differences.

Statistical significance was set at a two-tailed *p*-value < 0.05. A variance inflation factor (VIF) greater than 10 was taken to indicate the presence of multicollinearity. We conducted a power analysis for determining the minimum effective sample size required for our multivariate Cox regression analysis, following the method proposed by Riley et al. and utilizing the “pmsampsize” R package (18, 19). The key parameters selected for our analysis included: an event rate of 12%, representing the lowest integer value for in-hospital and ICU mortality rates observed in our cohort; a maximum of 17 candidate predictors, which was the highest number included in any of our constructed Cox regression models; and a Cox and Snell R-squared value set at 0.15. Our conservative estimate yielded a minimum effective sample size of 933, which is surpassed by the size of our actual sample. Analyses were executed using R software (version 4.2.3) and Python (version 3.9.13).

## Results

### CIPI and baseline characteristics

The selected manifest variables for EFA, NLR, SIRI, and SIII, exhibiting higher AUC across various time points, demonstrated strong correlations and multicollinearity, as shown by their high Pearson coefficients and variance inflation factors (VIFs; Figure 1). Following outlier removal, normality checks, and Box-Cox transformations, our data met the criteria for EFA (Figure 1). The resulting CIPI, derived from our EFA, is as follows: CIPI = 0.896 *SIII +0.797 *SIRI +0.923 *NLR. A total of 1,022 patients from the MIMIC-IV database were ultimately enrolled for analysis. The median age of the enrolled patients was 69 [59, 80] years, and 530 (51.9%) were men. The in-hospital mortality and ICU mortality rate were 17.4% and 12.8%, respectively. A total of 326 patients were selected from the MIMIC-III CareVue subset. The median age of the enrolled patients was 75 [59, 83] years, and 156 (47.9%) were men. The in-hospital mortality and ICU mortality rate were 30.4% and 19%, respectively.

Table 1 presents a comprehensive analysis of the variables in the study cohort, based on the CIPI quartiles. A progressive trend was observed across the quartiles for a wide array of variables. Specifically, the rates of congestive heart failure, sepsis3, RDW, PT, BUN, glucose, heart rate, respiratory rate, SAPSII, APSIII, hospital expire time, WBC, neutrophils, NLR, PLR, SIII, and SIRI increased incrementally from Q1 to Q4. Conversely, the median values of PPT, eosinophils, lymphocytes, LMR, PNR, and PWR showed a significant decrease from Q1 to Q4. The mortality rates within the hospital and ICU settings also escalated with higher CIPI scores, with in-hospital mortality rates rising from 10.5% in Q1 to 27.7% in Q4 and ICU mortality rates increasing from 8.2% in Q1 to 18.8% in Q4. These findings highlight the stepwise changes in clinical parameters across CIPI quartiles and underscore the potential role of CIPI scores in risk stratification among critically ill patients with cerebral infarction.

**Table 1:** Baseline characteristics of the study population in MIMIC-IV v 2.2.

Characteristic	Total (*n* = 1,022)	Q1 (*n* = 256)	Q2 (*n* = 255)	Q3 (*n* = 255)	Q4 (*n* = 256)	*P*-value
**Demographics**						
Age (years)	69.30 [59.21,80.05]	68.74 [59.66,78.70]	70.67 [60.53,81.81]	68.62 [57.87,78.87]	70.15 [59.31,81.05]	0.371
Male, *n*(%)	530 (51.9)	119 (46.5)	131 (51.4)	139 (54.5)	141 (55.1)	0.190
Married, *n*(%)	412 (40.3)	111 (43.4)	104 (40.8)	106 (41.6)	91 (35.5)	0.309
Race, *n*(%)						0.553
White	638 (62.4)	162 (63.3)	154 (60.4)	153 (60.0)	169 (66.0)	
Black	136 (13.3)	39 (15.2)	36 (14.1)	34 (13.3)	27 (10.5)	
Other	248 (24.3)	55 (21.5)	65 (25.5)	68 (26.7)	60 (23.4)	
Insurance, *n*(%)						0.086
Medicaid	67 (6.6)	15 (5.9)	12 (4.7)	17 (6.7)	23 (9.0)	
Medicare	480 (47.0)	115 (44.9)	121 (47.5)	110 (43.1)	134 (52.3)	
Other	475 (46.5)	126 (49.2)	122 (47.8)	128 (50.2)	99 (38.7)	
LOS hospital, (days)	9.97 [4.97,18.70]	7.60 [3.53,15.19]	10.73 [5.16,19.55]	10.92 [5.11,19.66]	11.68 [6.38,20.52]	<0.001
hospital mortality, *n*(%)	178 (17.4)	27 (10.5)	27 (10.6)	53 (20.8)	71 (27.7)	<0.001
LOS ICU, (days)	4.20 [1.89,9.95]	3.30 [0.69,7.64]	4.15 [1.80,10.62]	4.85 [2.34,11.70]	4.84 [2.17,10.86]	0.001
ICU mortality, *n*(%)	131 (12.8)	21 (8.2)	20 (7.8)	42 (16.5)	48 (18.8)	<0.001
**Vital signs**						
Heart rate (bpm)	79.98 [70.64,90.96]	78.03 [68.73,87.76]	79.04 [71.04,89.38]	79.81 [69.94,92.75]	82.92 [73.29,94.08]	0.001
Respiratory rate (bpm)	18.88 [16.91,21.38]	18.22 [16.46,20.58]	18.63 [16.99,21.01]	19.13 [16.94,21.82]	19.45 [17.80,21.92]	<0.001
SBP (mmHg)	127.17 [113.09,141.82]	126.12 [113.89,141.76]	129.20 [116.42,141.79]	123.56 [110.37,140.83]	127.99 [112.30,143.43]	0.174
DBP (mmHg)	67.22 [58.70,76.36]	68.36 [61.19,78.00]	67.88 [58.97,76.25]	65.39 [57.71,74.88]	66.73 [58.46,75.62]	0.024
MBP (mmHg)	84.04 [74.93,93.42]	85.91 [76.02,94.70]	85.71 [75.36,94.57]	82.20 [72.83,92.41]	83.29 [74.85,92.88]	0.042
Temperature (°C)	36.87 [36.66,37.12]	36.79 [36.63,37.03]	36.86 [36.68,37.09]	36.94 [36.67,37.16]	36.91 [36.68,37.21]	0.002
SpO2 (%)	97.18 [95.88,98.57]	97.18 [96.07,98.61]	97.28 [95.90,98.66]	96.99 [95.75,98.43]	97.29 [95.79,98.56]	0.745
**Laboratory parameters**						
CIPI	−0.02[−0.69,0.66]	−1.11[−1.42,-0.86]	−0.35[−0.51,-0.21]	0.31 [0.15,0.48]	1.20 [0.95,1.51]	<0.001
PLR	144.69 [95.29,22.10]	89.44 [62.54,116.96]	128.76 [93.57,167.40]	170.69 [121.61,217.65]	259.46 [183.24,341.30]	<0.001
SIRI	3.01 [1.54,6.02]	1.03 [0.57,1.52]	2.37 [1.73,3.27]	4.10 [2.87,5.78]	8.71 [5.96,11.32]	<0.001
SIII	1077.84 [560.90,1906.41]	337.66 [245.34,515.87]	843.50 [654.83,1022.53]	1411.87 [1130.13,1721.11]	2830.39 [2131.95,3637.40]	<0.001
LMR	2.47 [1.57,3.77]	4.07 [3.09,6.47]	2.75 [2.01,3.90]	2.06 [1.47,3.24]	1.37 [0.90,2.00]	<0.001
PWR	20.38 [14.41,27.94]	25.08 [16.92,35.01]	21.57 [16.74,28.92]	19.03 [13.50,24.70]	17.25 [12.56,22.89]	<0.001
PNR	27.21 [18.38,39.81]	41.63 [27.94,61.44]	30.13 [21.94,41.05]	24.15 [15.92,31.51]	19.94 [14.51,26.45]	<0.001
NLR	5.54 [3.19,9.33]	2.17 [1.60,2.76]	4.18 [3.54,4.92]	7.08 [6.08,8.47]	12.95 [10.32,16.13]	<0.001
WBC (10^9^/L)	9.90 [7.62,13.10]	7.60 [5.70,9.43]	9.20 [7.10,11.80]	10.80 [8.35,13.55]	13.00 [10.40,15.83]	<0.001
Neutrophils (10^9^/L)	7.40 [5.27,10.64]	4.56 [3.28,5.98]	6.61 [5.12,8.24]	8.46 [6.66,11.02]	11.20 [8.90,13.71]	<0.001
Lymphocytes (10^9^/L)	1.39 [0.89,1.99]	2.07 [1.56,2.78]	1.58 [1.23,2.11]	1.23 [0.88,1.67]	0.85 [0.63,1.21]	<0.001
Monocytes (10^9^/L)	0.58 [0.38,0.83]	0.48 [0.34,0.67]	0.58 [0.40,0.84]	0.58 [0.39,0.89]	0.65 [0.43,0.91]	<0.001
Basophils (10^9^/L)	0.03 [0.01,0.05]	0.03 [0.02,0.05]	0.03 [0.02,0.05]	0.03 [0.01,0.05]	0.03 [0.01,0.04]	0.004
Eosinophils (10^9^/L)	0.06 [0.02,0.15]	0.12 [0.05,0.22]	0.09 [0.03,0.18]	0.04 [0.01,0.09]	0.03 [0.01,0.09]	<0.001
RBC (10^12^/L)	3.95 [3.33,4.48]	3.98 [3.26,4.49]	3.95 [3.38,4.52]	3.91 [3.37,4.46]	3.95 [3.39,4.46]	0.939
RDW (fL)	14.10 [13.20,15.40]	13.70 [13.00,14.90]	14.10 [13.20,15.40]	14.20 [13.30,15.70]	14.30 [13.30,15.60]	0.002
Hematocrit (%)	35.50 [30.33,39.90]	35.70 [29.78,40.12]	35.80 [30.40,40.15]	35.50 [30.35,39.50]	35.25 [30.60,39.80]	0.986
Hemoglobin (g/dL)	11.60 [9.90,13.30]	11.65 [9.75,13.40]	11.60 [10.00,13.40]	11.70 [9.70,13.25]	11.60 [9.90,13.33]	0.997
Platelet (10^9^/L)	200.50 [151.00,260.00]	183.00 [134.00,231.25]	206.00 [154.50,251.50]	200.00 [149.00,258.50]	215.50 [167.00,284.25]	<0.001
INR	1.20 [1.10,1.38]	1.10 [1.00,1.30]	1.10 [1.00,1.30]	1.20 [1.10,1.40]	1.20 [1.10,1.40]	<0.001
PT(s)	12.80 [11.70,14.80]	12.32 [11.40,14.50]	12.60 [11.40,14.50]	13.20 [11.95,15.80]	13.30 [12.10,14.93]	<0.001
PTT(s)	29.10 [26.20,33.40]	29.60 [26.92,34.42]	29.30 [25.75,33.10]	29.00 [26.40,34.10]	28.25 [26.00,31.95]	0.031
Aniongap (mEq/L)	15 [13.00,17.00]	14 [13.00,16.00]	15 [13.00,17.00]	15 [13.00,17.00]	15 [13.35,18.00]	<0.001
Bicarbonate (mmol/L)	23.00 [21.00,25.60]	24.00 [22.00,26.00]	23.20 [21.00,26.00]	23.00 [21.00,25.00]	23.00 [21.00,25.00]	0.02
BUN (mg/dL)	19.00 [13.00,28.00]	17.00 [12.00,24.05]	18.00 [13.00,26.00]	20.00 [14.00,30.00]	22.00 [15.00,32.00]	<0.001
Creatinine (mg/dL)	1.00 [0.80,1.40]	0.90 [0.70,1.20]	1.00 [0.80,1.30]	1.00 [0.80,1.60]	1.10 [0.80,1.60]	0.001
Glucose (mg/dL)	123.00 [130.00,158.00]	111.00 [94.00,132.55]	121.00 [101.00,152.50]	126.00 [106.00,158.90]	139.50 [115.00,197.00]	<0.001
**Comorbidities, *n* (%)**						
Myocardial infarction	193 (18.9)	42 (16.4)	39 (15.3)	46 (18.0)	66 (25.8)	0.01
Congestive heart failure	292 (28.6)	55 (21.5)	68 (26.7)	75 (29.4)	94 (36.7)	0.002
Diabetes mellitus	359 (35.1)	85 (33.2)	89 (34.9)	90 (35.3)	95 (37.1)	0.834
Renal disease	227 (22.2)	48 (18.8)	54 (21.2)	60 (23.5)	65 (25.4)	0.297
Liver disease	23 (2.3)	8 (3.1)	4 (1.6)	6 (2.4)	5 (2.0)	0.672
Malignancy	93 (9.1)	23 (9.0)	19 (7.5)	25 (9.8)	26 (10.2)	0.72
CCI	6.00 [4.00,8.00]	6.00 [4.00,8.00]	6.00 [5.00,9.00]	6.00 [4.00,9.00]	7.00 [5.00,9.00]	0.451
Sepsis3, *n* (%)	490 (47.9)	97 (37.9)	117 (45.9)	120 (47.1)	156 (60.9)	<0.001
**Prognosis scores**						
GCS	15.00 [14.00,15.00]	15.00 [14.75,15.00]	15.00 [14.00,15.00]	15.00 [14.00,15.00]	15.00 [14.00,15.00]	0.067
SIRS	2.00 [2.00,3.00]	2.00 [1.00,3.00]	2.00 [2.00,3.00]	2.00 [2.00,3.00]	2.00 [2.00,3.00]	<0.001
SOFA	4.00 [2.00,6.00]	3.00 [1.00,6.00]	4.00 [2.00,6.00]	4.00 [2.00,7.00]	4.00 [2.00,6.00]	<0.001
SAPSII	35.00 [27.00,43.75]	31.00 [24.00,41.00]	34.00 [25.00,43.00]	36.00 [28.00,45.00]	38.00 [29.75,45.00]	<0.001
OASIS	32.00 [26.00,38.00]	29.00 [24.00,35.00]	32.00 [26.00,37.00]	33.00 [28.00,40.00]	33.00 [28.00,40.00]	<0.001
LODS	4.00 [2.00,6.00]	3.00 [1.00,5.00]	3.00 [2.00,6.00]	4.00 [2.00,7.00]	4.00 [2.00,7.00]	<0.001
APSIII	38.00 [29.00,54.00]	34.00 [24.75,48.25]	37.00 [28.00,51.00]	43.00 [31.50,59.00]	44.00 [32.00,57.00]	<0.001

LOS, length of stay; CIPI, comprehensive inflammatory prognostic index; PLR, platelet to lymphocyte ratio; SIRI, systemic inflammation response index; SIII, systemic immune inflammation index; LMR, lymphocyte to monocyte ratio; PWR, platelet to white blood cell ratio; PNR, platelet to neutrophil ratio; NLR, platelet to lymphocyte ratio; WBC, white blood cells; RBC, red blood cells; RDW, red blood cell distribution width; INR, international normalized ratio; PT, prothrombin time; PTT, partial thromboplastin time; BUN, blood urea nitrogen; CCI, Charlson comorbidity index; GCS, Glasgow coma score; SIRS, systemic inflammatory response syndrome; SOFA, sequential organ failure assessment; SAPSII, simplified acute physiology score II; OASIS, Oxford acute severity of illness score; LODS, logistic organ dysfunction score; APSIII, acute physiology score III.

### Survival curves and linearity assessment

Kaplan–Meier survival curves were plotted for the four groups defined by CIPI quartiles and compared using the log-rank test. The *p*-values of 0.00011 and 0.012, displayed on the survival plots (Supplementary Figures 1A,B), denote statistically significant differences in survival distributions across these quartiles. This highlights the impact of CIPI on both patient in-hospital mortality and ICU mortality. According to the restricted cubic spline plots and AIC comparisons (*p* = 0.051 and *p* = 0.132), CIPI appears to have a linear relationship with both in-hospital and ICU mortality rates (Supplementary Figures 1C,D).

### Univariate Cox regression analysis

In the MIMIC-IV dataset (Table 2), all four indices (CIPI, SIRI, SIII, and NLR) showed significant HR with both in-hospital and ICU mortality when treated as continuous variables. In quartile analysis, all indices, excluding SIII, exhibited significant HR differences between Q1 and Q4, along with a significant increasing trend from Q1 to Q4. Notably, CIPI also revealed a significant HR difference between Q1 and Q3. When we applied the same methods used in the MIMIC-IV dataset to the MIMIC-III CareVue subset (Table 2), only CIPI demonstrated statistically significant HR in predicting both in-hospital and ICU mortality when treated as a continuous variable. Analyzed as quartiles, all indices lacked significant trends from Q1 to Q4, likely due to the smaller sample size. For in-hospital mortality, only CIPI demonstrated a significant HR difference between Q1 and Q4. For ICU mortality, such a significant HR difference was seen only in CIPI and SIII between Q1 and Q3. The results consistently emphasized the superior predictive performance and robustness of CIPI over its individual manifest variables used in the EFA, in both the original dataset and external validation, thus underscoring the value of consolidating these variables into a single index.

**Table 2:** Univariate Cox regression analysis for CIPI, SIRI, SIII, and NLR.

	CIPI		SIRI		SIII		NLR	
	HR (95% CI)	*P*-value	HR (95% CI)	*P*-value	HR (95% CI)	*P*-value	HR (95% CI)	*P*-value
**In-hospital mortality (MIMIC-IV database)**								
Cont.	1.422 (1.213,1.668)	<0.001	1.252 (1.124,1.395)	<0.001	1.047 (1.021,1.074)	<0.001	1.311 (1.158,1.485)	<0.001
Quartile	*P* for trend:<0.001		*P* for trend: <0.001		*P* for trend:0.001		*P* for trend:<0.001	
Q1	Reference		Reference		Reference		Reference	
Q2	0.812 (0.479,1.394)	0.459	1.111 (0.664,1.858)	0.689	0.620 (0.367,1.047)	0.074	0.869 (0.504,1.499)	0.614
Q3	1.607 (1.008,2.561)	0.046	1.341 (0.828,2.172)	0.234	1.065 (0.690,1.644)	0.777	1.475 (0.896,2.428)	0.127
Q4	1.980 (1.270,3.089)	0.003	2.380 (1.532,3.696)	<0.001	1.648 (1.105,2.457)	0.014	2.228 (1.396,3.556)	<0.001
**ICU mortality (MIMIC-IV database)**								
Cont.	1.369 (1.133,0.655)	0.001	1.203 (1.062,1.363)	0.004	1.036 (1.006,1.067)	0.019	1.304 (1.122,1.516)	<0.001
Quartile	*P* for trend:0.006		*P* for trend: 0.002		*P* for trend:0.076		*P* for trend:<0.001	
Q1	Reference		Reference		Reference		Reference	
Q2	0.748 (0.405,1.382)	0.354	1.204 (0.668,2.169)	0.537	0.735 (0.417,1.297)	0.289	0.824 (0.346,1.556)	0.550
Q3	1.418 (0.838,2.397)	0.193	1.245 (0.711,2.178)	0.443	0.956 (0.579,1.578)	0.859	1.288 (0.723,2.292)	0.390
Q4	1.651 (1.002,2.762)	0.049	2.147 (1.282,3.595)	0.004	1.389 (0.871,2.217)	0.168	1.946 (1.125,3.367)	0.017
**In-hospital mortality (MIMIC-III CareVue subset: external validation)**								
Cont.	1.232 (1.004,1.511)	0.045	1.189 (0.997,1.417)	0.054	1.021 (0.998,1.044)	0.071	1.153 (0.999,1.329)	0.051
Quartile	*P* for trend:0.068		*P* for trend:0.132		*P* for trend:0.067		*P* for trend:0.116	
Q1	Reference		Reference		Reference		Reference	
Q2	1.710 (0.921,3.178)	0.090	1.189 (0.997,1.417)	0.887	1.367 (0.737,2.535)	0.321	1.726 (0.921,3.235)	0.089
Q3	1.742 (0.927,3.272)	0.085	1.049 (0.575,1.912)	0.877	1.780 (0.983,3.222)	0.057	1.792 (0.947,3.390)	0.073
Q4	1.848 (1.010,3.381)	0.046	1.521 (0.872,2.653)	0.140	1.651 (0.919,2.996)	0.093	1.748 (0.939,3.254)	0.078
**ICU mortality (MIMIC-III CareVue subset: external validation)**								
Cont.	1.305 (1.003,1.699)	0.047	1.214 (0.964,1.529)	0.099	1.026 (0.997,1.056)	0.078	1.205 (1.003,1.446)	0.046
Quartile	*P* for trend:0.063		*P* for trend:0.309		*P* for trend:0.098		*P* for trend:0.078	
Q1	Reference		Reference		Reference		Reference	
Q2	1.929 (0.792,4.697)	0.148	0.998 (0.456,2.186)	0.997	1.371 (0.586,3.210)	0.467	1.845 (0.758,4.488)	0.177
Q3	2.677 (1.108,6.467)	0.029	1.088 (0.491,2.411)	0.836	2.700 (1.234,5.910)	0.013	2.244 (0.924,5.451)	0.074
Q4	2.278 (0.961,5.402)	0.062	1.388 (0.667,2.886)	0.380	1.711 (0.764,3.831)	0.192	2.223 (0.942,5.246)	0.068

### Multivariate Cox regression analysis

As seen in Table 3, our results indicate that the CIPI, as a continuous variable, consistently exerted significant predictive power for both in-hospital and ICU mortality across all four models, emphasizing its independence and robustness as a prognostic factor. Upon categorization into quartiles, a significant trend in CIPI signaled a dose–response relationship with mortality risk. Particularly for in-hospital mortality, higher CIPI quartiles were significantly correlated with increased risk across all models. This, however, was not mirrored in ICU mortality, where interquartile comparisons did not consistently show statistical significance. This suggests that the CIPI quartiles might not be able to capture the nuances of ICU mortality risk effectively. To address this, we transformed CIPI into a trichotomy, which revealed that higher CIPI values were indeed significantly associated with elevated ICU mortality risk. These findings underscore the potential utility of CIPI as a nuanced prognostic tool for critically ill patients with cerebral infarction.

**Table 3:** Performance of CIPI in multivariate Cox analysis.

	Model 1		Model 2		Model 3		Model 4	
	HR (95% CI)	*P*-value	HR (95% CI)	*P*-value	HR (95% CI)	*P*-value	HR (95% CI)	*P*-value
**In-hospital mortality**								
Cont.	1.432 (1.219,1.682)	<0.001	1.419 (1.209,1.667)	<0.001	1.337 (1.126,1,588)	<0.001	1.357 (1.145,1.609)	<0.001
Quartile	*P* for trend:<0.001		*P* for trend: <0.001		*P* for trend:0.003		*P* for trend: 0.001	
Q1 (256)	Reference		Reference		Reference		Reference	
Q2 (255)	0.783 (0.457,1.339)	0.371	0.822 (0.477,1.416)	0.480	0.758 (0.436,1.318)	0.326	0.737 (0.430,1.261)	0.265
Q3 (255)	1.615 (1.012,2.579)	0.045	1.719 (1.069,2.763)	0.025	1.445 (0.886,2.358)	0.140	1.489 (0.926,2.393)	0.101
Q4 (256)	1.955 (1.248,3.064)	0.003	1.908 (1.202,3.027)	0.006	1.618 (1.002,2.611)	0.049	1.656 (1.042,2.631)	0.033
**ICU mortality (Quartile)**								
Cont.	1.387 (1.145,1.679)	<0.001	1.374 (1.132,1.666)	0.001	1.367 (1.112,1.679)	0.003	1.394 (1.148,1.693)	<0.001
Quartile	*P* for trend:0.005		*P* for trend: 0.008		*P* for trend:0.016		*P* for trend:0.005	
Q1 (256)	Reference		Reference		Reference		Reference	
Q2 (255)	0.699 (0.376,1.300)	0.258	0.754 (0.403,1.409)	0.376	0.711 (0.378,1.336)	0.289	0.673 (0.361,1.256)	0.214
Q3 (255)	1.421 (0.383,2.410)	0.192	1.567 (0.916,2.681)	0.101	1.476 (0.846,2.575)	0.170	1.468 (0.865,2.491)	0.155
Q4 (256)	1.630 (0.966,2.751)	0.067	1.602 (0.930,2.758)	0.089	1.542 (0.876,2.713)	0.133	1.580 (0.933,2.676)	0.089
**ICU mortality (trichotomy)**								
Quartile	*P* for trend:0.003		*P* for trend: 0.005		*P* for trend:0.009		*P* for trend:0.004	
Q1 (341)	Reference		Reference		Reference		Reference	
Q2 (340)	1.150 (0.696,1.900)	0.586	1.244 (0.745,2.077)	0.403	1.204 (0.709,2.045)	0.492	1.114 (0.671,1.848)	0.676
Q3 (341)	1.882 (1.192,2.974)	0.007	1.898 (1.182,3.046)	0.008	1.866 (1.125,3.093)	0.016	1.859 (1.171,2.952)	0.009

Model 1: Adjusted for CIPI, age, gender, insurance, race, marital status. Model 2: Adjusted for myocardial infarction, congestive heart failure, chronic pulmonary disease, rheumatic disease, peptic ulcer disease, diabetes, renal disease, malignant cancer, severe liver disease, Charlson comorbidity index, sepsis3, and covariates with statistically significant HR from the previous model. Model 3: Adjusted for hematocrit, hemoglobin, platelet count, RDW, RBC, INR, PT, PTT, anion gap, bicarbonate, BUN, creatinine, glucose, and covariates with statistically significant HR from the previous model. Model 4: Adjusted for 24-h mean values of heart rate, systolic blood pressure, diastolic blood pressure, mean blood pressure, respiratory rate, body temperature, SpO2, and significant covariates from the previous model.

**Figure 2:**
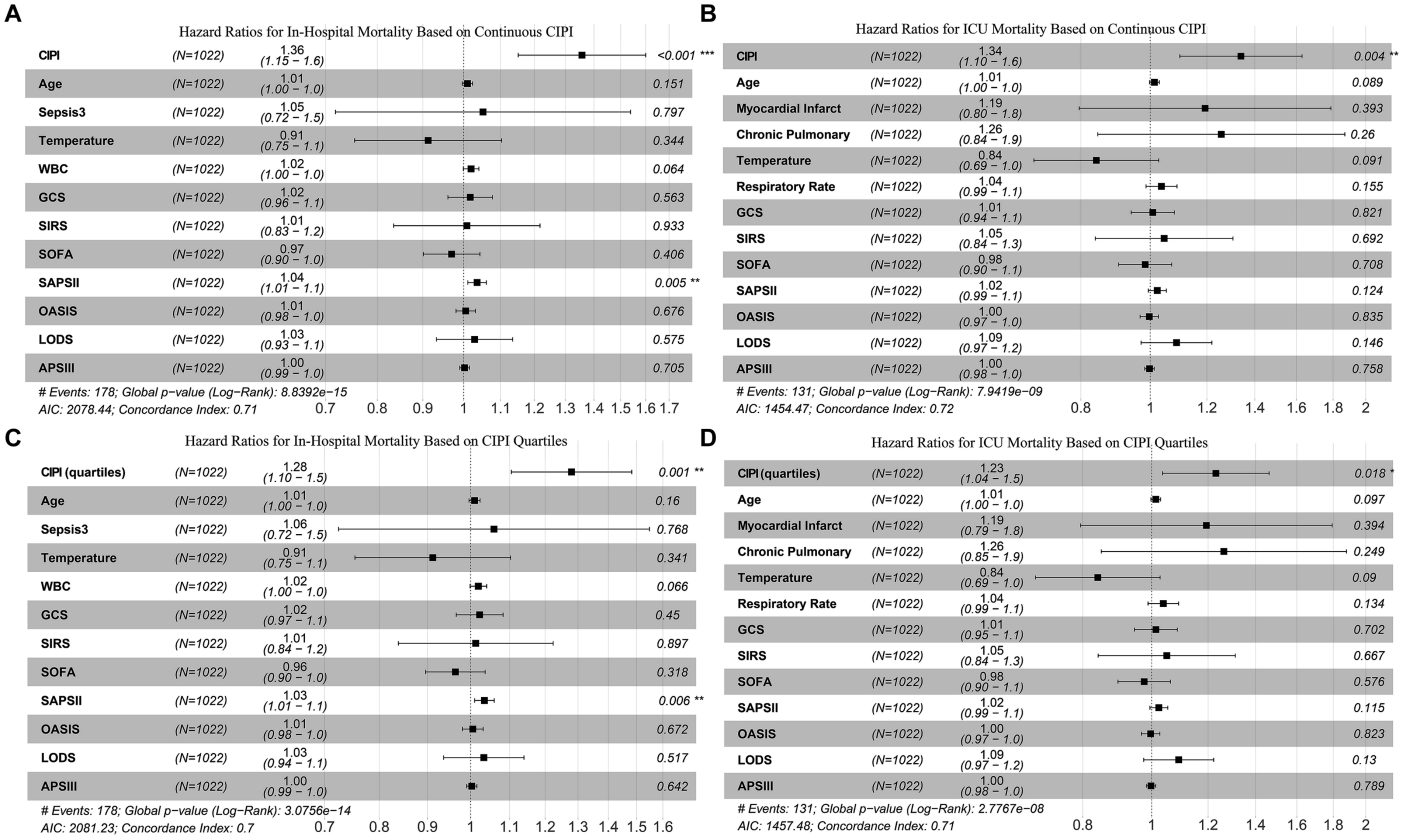
Multivariate Cox analysis confirms that CIPI is an independent prognostic factor for both in-hospital mortality (**A** and **C**) and ICU mortality (**B** and **D**).

Utilizing all independent prognostic factors identified in above models, and corroborated through VIF analysis to avoid multicollinearity with well-recognized ICU prognostic indicators, we formulated our final model, as demonstrated in Figure 2. The results showed that, for in-hospital mortality prediction, only CIPI and SAPSII surfaced as independent prognostic factors. On the other hand, for ICU mortality, CIPI alone maintained its prognostic significance. The results of including CIPI as a four-category graded variable in the model align with those observed when it is treated as a continuous variable. These findings strongly underscore the unique clinical value of CIPI as an independent factor in predicting mortality outcomes.

### Subgroup and interaction analysis

For in-hospital mortality (Supplementary Figure 2A), all subgroups (except those aged under 60) displayed statistically significant HR values. The risk change associated with each increase in CIPI varied among these subgroups. For instance, in patients where cerebral infarction was the first diagnosis, each increase in CIPI resulted in a 2.041-fold higher death risk [HR (95% CI) 2.041 (1.501–2.776)]. However, when cerebral infarction was not the first diagnosis, the risk was 1.237-fold [HR (95% CI) 1.237 (1.026–1.491)]. For patients with severe infections, the risk was 1.226-fold [HR (95% CI) 1.226 (1.023–1.470)]. Without severe infections, the risk more than doubled to 2.092-fold [HR (95% CI) 2.092 (1.517–2.886)]. These results reaffirm the CIPI’s robust independent prognostic power, given that the interaction *p*-values were above 0.05 for all variables, except sepsis (*p* = 0.002). For ICU mortality (Supplementary Figure 2B), the HR values for CIPI were not statistically significant in several subgroups. However, in all other subgroups, the HR values were significant, proving that the CIPI remains a strong prognostic tool even after controlling for these factors. The absence of significant interaction with these subgroups confirms CIPI’s independent predictive value.

### Time-dependent survival analysis

CIPI demonstrates superior predictive performance over several existing prognostic indicators for in-hospital mortality across various time points (Table 4). Notably, the CIPI consistently outperforms the GCS in terms of AUC at all analyzed time points except for the first day, and surpasses the SIRS and SOFA in AUC at day 8. In the context of ICU mortality, CIPI exceeds the GCS in AUC for mortality prediction at 2, 4, 6, and 8 days, but the OASIS performs better within the first day (Table 4). There is no significant difference in AUC between the CIPI and other established prognostic indicators at the remaining time points, for either in-hospital or ICU mortality.

**Table 4:** Comparison of time-dependent survival analysis AUC values.

In-hospital mortality	Day 1		Day 2		Day 4		Day 6	
	AUC (95% CI)	*P*	AUC (95% CI)	*P*	AUC (95% CI)	*P*-value	AUC (95% CI)	*P*
CIPI	0.689 (0.569,0.809)		0.753 (0.674,0.827)		0.672 (0.586,0.746)		0.650 (0.574,0.712)	
CIPI-EX	0.795 (0.697,0.888)		0.672 (0.529,0.817)		0.631 (0.519,0.723)		0.585 (0.484,0.659)	
GCS	0.655 (0.641,0.670)	0.588	0.567 (0.474,0.666)	0.009	0.480 (0.401,0.560)	<0.001	0.489 (0.424,0.552)	0.001
SIRS	0.443 (0.196,0.690)	0.124	0.701 (0.561,0.842)	0.552	0.623 (0.523,0.706)	0.426	0.599 (0.506,0.662)	0.283
SOFA	0.787 (0.632,0.943)	0.437	0.635 (0.479,0.784)	0.201	0.605 (0.492,0.694)	0.410	0.594 (0.490,0.654)	0.226
SAPSII	0.765 (0.610,0.921)	0.506	0.683 (0.545,0.814)	0.367	0.702 (0.620,0.766)	0.634	0.678 (0.600,0.733)	0.634
OASIS	0.786 (0.676,0.897)	0.293	0.776 (0.683,0.864)	0.721	0.770 (0.708,0.832)	0.064	0.729 (0.665,0.790)	0.074
LODS	0.810 (0.659,0.962)	0.338	0.693 (0.558,0.822)	0.468	0.686 (0.599,0.756)	0.850	0.654 (0.567,0.707)	0.915
APSIII	0.756 (0.589,0.922)	0.538	0.715 (0.585,0.840)	0.613	0.684 (0.586,0.764)	0.890	0.657 (0.570,0.722)	0.951
**In-hospital mortality**	**Day 8**		**Day 12**		**Day 16**		**Day 20**	
	**AUC (95% CI)**	***P***	**AUC (95% CI)**	***P***	**AUC (95% CI)**	***P*-value**	**AUC (95% CI)**	***P***
CIPI	0.663 (0.599,0.721)		0.609 (0.545,0.673)		0.593 (0.523,0.644)		0.584 (0.515,0.638)	
CIPI-EX	0.570 (0.464,0.642)		0.563 (0.452,0.628)		0.544 (0.436,0.622)		0.588 (0.461,0.660)	
GCS	0.486 (0.427,0.537)	<0.001	0.462 (0.398,0.501)	<0.001	0.502 (0.447,0.539)	0.014	0.495 (0.442,0.540)	0.027
SIRS	0.565 (0.477,0.612)	0.014	0.565 (0.484,0.603)	0.141	0.568 (0.495,0.607)	0.440	0.563 (0.493,0.608)	0.549
SOFA	0.574 (0.477,0.619)	0.032	0.573 (0.475,0.601)	0.140	0.569 (0.486,0.608)	0.436	0.572 (0.481,0.608)	0.501
SAPSII	0.648 (0.567,0.670)	0.493	0.662 (0.599,0.710)	0.311	0.643 (0.577,0.688)	0.247	0.641 (0.556,0.676)	0.368
OASIS	0.696 (0.631,0.747)	0.497	0.670 (0.598,0.713)	0.280	0.622 (0.549,0.667)	0.548	0.605 (0.520,0.645)	0.892
LODS	0.653 (0.576,0.697)	0.602	0.621 (0.530,0.651)	0.676	0.616 (0.538,0.652)	0.782	0.617 (0.525,0.647)	0.835
APSIII	0.644 (0.562,0.694)	0.498	0.624 (0.543,0.668)	0.939	0.607 (0.538,0.656)	0.761	0.601 (0.521,0.845)	0.889
**ICU mortality**	**Day 1**		**Day 2**		**Day 4**		**Day 6**	
	**AUC (95% CI)**	***P***	**AUC (95% CI)**	***P***	**AUC (95% CI)**	***P***	**AUC (95% CI)**	***P***
CIPI	0.651 (0.529,0.771)		0.718 (0.624,0.798)		0.609 (0.514,0.696)		0.601 (0.512,0.668)	
CIPI-EX	0.771 (0.623,0.912)		0.622 (0.498,0.753)		0.559 (0.444,657)		0.561 (0.445,0.649)	
GCS	0.532 (0.410,0.656)	0.111	0.527 (0.422,0.625)	0.003	0.454 (0.361,0.519)	0.004	0.485 (0.408,0.536)	0.015
SIRS	0.582 (0.403,0.762)	0.576	0.685 (0.570,0.806)	0.753	0.628 (0.540,0.710)	0.739	0.599 (0.513,0.671)	0.970
SOFA	0.783 (0.646,0.925)	0.181	0.649 (0.490,0.781)	0.434	0.571 (0.454,0.651)	0.444	0.579 (0.485,0.665)	0.809
SAPSII	0.764 (0.612,0.918)	0.222	0.696 (0.558,0.814)	0.763	0.685 (0.608,0.748)	0.213	0.682 (0.617,0.760)	0.066
OASIS	0.824 (0.745,0.909)	0.032	0.765 (0.668,0.845)	0.528	0.698 (0.621,0.773)	0.133	0.678 (0.610,0.761)	0.071
LODS	0.787 (0.634,0.947)	0.167	0.703 (0.564,0.813)	0.796	0.649 (0.548,0.706)	0.733	0.635 (0.549,0.705)	0.518
APSIII	0.776 (0.637,0.918)	0.175	0.723 (0.595,0.840)	0.937	0.638 (0.533,0.714)	0.777	0.628 (0.540,0.712)	0.559
**ICU mortality**	**Day 8**		**Day 12**		**Day 16**		**Day 20**	
	**AUC (95% CI)**	***P***	**AUC (95% CI)**	***P***	**AUC (95% CI)**	***P***	**AUC (95% CI)**	***P***
CIPI	0.604 (0.535,0.685)		0.561 (0.484,0.638)		0.577 (0.523,0.673)		0.563 (0.472,0.634)	
CIPI-EX	0.588 (0.453,0.662)		0.608 (0.507,0.722)		0.547 (0.412,0.664)		0.576 (0.430,0.706)	
GCS	0.495 (0.428,0.540)	0.008	0.503 (0.439,0.546)	0.143	0.519 (0.465,0.571)	0.076	0.506 (0.453,0.574)	0.413
SIRS	0.563 (0.490,0.634)	0.377	0.550 (0.454,0.592)	0.460	0.543 (0.462,0.606)	0.213	0.545 (0.427,0.580)	0.365
SOFA	0.565 (0.487,0.644)	0.462	0.563 (0.479,0.625)	0.873	0.556 (0.482,0.632)	0.474	0.552 (0.468,0.632)	0.969
SAPSII	0.643 (0.580,0.720)	0.461	0.646 (0.575,0.706)	0.135	0.628 (0.552,0.693)	0.648	0.620 (0.518,0.680)	0.434
OASIS	0.639 (0.584,0.726)	0.355	0.613 (0.545,0.685)	0.285	0.571 (0.511,0.664)	0.841	0.556 (0.476,0.645)	0.889
LODS	0.634 (0.579,0.714)	0.492	0.598 (0.509,0.653)	0.715	0.594 (0.511,0.655)	0.775	0.588 (0.497,0.661)	0.652
APSIII	0.615 (0.546,0.690)	0.878	0.590 (0.509,0.652)	0.720	0.580 (0.506,0.655)	0.739	0.573 (0.476,0.641)	0.912

Further, the CIPI underwent external validation, with results illustrated in Table 4. This validation strengthens the evidence of CIPI’s robustness and reliability as a prognostic tool.

## Discussion

This study introduces CIPI and validates its role as an independent prognostic factor for critically ill patients with cerebral infarction. Its predictive capacity is comparable to that of established prognostic indicators. However, the CIPI offers notable advantages in terms of simplicity and operational ease. It relies solely on the results of routine blood tests. With a programmed calculation using these readily available values, clinicians can quickly and effortlessly obtain the CIPI, simplifying the prognostic process significantly.

In our study, we diligently improved our research methodology to ensure robust and accurate results. We used the MIMIC-III CareVue subset as an independent validation set, unlike previous studies that combined unfiltered data from MIMIC-III and MIMIC-IV (20–24), which can lead to data duplication and affect the stability and accuracy of results. In most research on ischemic stroke patients using the MIMIC database, the selection of cases is overly broad. Many studies indiscriminately include all patients coded under ICD-9: 433, ICD-9: 434, ICD-9: 436, and ICD-10: I63 (25–28). However, half of these specific sub-codes, such as 433.00: Occlusion and stenosis of the basilar artery without mention of cerebral infarction, 433.10: Occlusion and stenosis of the carotid artery without mention of cerebral infarction, 433.20: Occlusion and stenosis of the vertebral artery without mention of cerebral infarction, and 433.30: Occlusion and stenosis of multiple and bilateral precerebral arteries without mention of cerebral infarction, do not necessarily diagnose an ischemic stroke. Instead, they signify susceptibility factors for such a stroke. Consequently, this indiscriminate approach to case selection creates a significant selection bias. In statistical terms, this is problematic because it can lead to an overestimation of the associations between the supposed risk factors and ischemic stroke outcomes. Such overestimation can distort the true relationships under study and result in spurious conclusions. By strictly selecting records based on a cerebral infarction diagnosis, we minimized such selection bias, hence enhancing the accuracy and validity of our study. Due to the aforementioned bias in case selection, the independent prognostic utility of many indices warrants reevaluation. For instance, while numerous studies have validated NLR, SII, and SIRI as effective prognostic indicators, our stringent selection criteria and univariate Cox regression analysis results revealed that the performance of these indices individually was not as effective, particularly when compared with our newly proposed CIPI index in the MIMIC-III CareVue subset.

The robustness of CIPI as an independent prognostic indicator was extensively validated in our study. Following rigorous variable selection and multicollinearity tests, we constructed a final multivariate Cox regression model that included several officially recognized prognostic indices from the MIMIC database, such as GCS, SIRS, SOFA, SAPSII, OASIS, LODS, and APSIII (16, 29), as illustrated in Figure 2. The results revealed that CIPI and SAPSII were the only independent predictors of in-hospital mortality. Moreover, CIPI emerged as the unique independent predictor for ICU mortality. This indicates that many prognostic indicators, although effective, may overlap in their predictive functions for critically ill cerebral infarction patients and lose their individual prognostic utility when combined. The CIPI we introduced is a latent variable derived from EFA of NLR, SIII, and SIRI, encapsulating their combined contributions to critically ill patient prognosis in cerebral infarction. The composition of NLR, SIII, and SIRI share common elements, such as neutrophils and lymphocytes, yet each has its focus: SIII on platelets, and SIRI on monocytes (9, 30, 31). This composition reflects their inherent high correlations and unique characteristics. Together, they capture various aspects of the systemic inflammatory response and coagulation process, key factors influencing outcomes in cerebral infarction patients (32). Inflammation is a key mechanism in stroke, and the post-stroke inflammatory response plays a vital role in secondary brain injury and activation of coagulation (33, 34). The disruption of normal coagulation processes after cerebral infarction can lead to exacerbated ischemic damage in the brain, and also activate multiple inflammatory pathways (35). The changes in peripheral blood indicators like neutrophils, lymphocytes, monocytes, and platelets are sensitive markers of the body’s cascade response to cerebral infarction, especially in the presence of severe complications and comorbidities (36, 37). By conducting EFA on NLR, SIII, and SIRI, CIPI maintains their common features while enhancing their unique contributions. This underpins CIPI’s independence compared to traditional ICU prognostic indicators. Furthermore, our confirmation of its linear relationship with mortality rates, along with its resilience to interaction effects, strengthens our belief in CIPI’s potential for clinical application as an independent prognostic tool.

In our time-dependent AUC analysis, we observed that no existing prognostic indices from the MIMIC database, such as GCS, SIRS, SOFA, SAPSII, OASIS, LODS, and APSIII (16, 29), statistically significantly outperformed CIPI at all analyzed time points. This indicates that, overall, CIPI’s ability to predict in-hospital and ICU mortality among critically ill cerebral infarction patients is on par with these established indices. This equivalency in performance could be attributed to the fact that these indices, including CIPI, reflect patient prognosis from different perspectives, as supported by references (38–44). Given the complexity and rapid disease progression in ICU settings, it is challenging to identify a single prognostic indicator that completely surpasses others in all dimensions of patient care. This highlights the multifaceted nature of critical care prognosis, where each indicator provides unique insights without statistically outperforming the others. Hence, the deep research value of a prognostic indicator in such a context lies significantly in its accessibility and ease of evaluation. Recent studies have also been focused on finding simple indices for predicting outcomes in critically ill cerebral infarction patients. For instance, Zhao et al. reported AUC values of 0.552, 0.644, and 0.541 for NLR, neutrophil to albumin ratio, and red cell distribution width to albumin ratio, respectively, in predicting 30-day mortality in critical stroke patients (45). Jhou et al. found that plasma anion gap predicted in-hospital mortality in ICU acute ischemic stroke patients with an AUC of 0.631 (46). Chen et al. used the triglyceride glucose index to predict in-hospital mortality in critical cerebrovascular disease patients, achieving an AUC of 0.610 (47). Our CIPI showed AUC values greater than 0.6 for predicting both in-hospital and ICU mortality at multiple time points, with an AUC of 0.753 for predicting in-hospital mortality and 0.718 for ICU mortality on the second day of ICU admission. This predictive ability is, to a certain extent, superior to the aforementioned indices. Additionally, CIPI offers a significant advantage as it is derived entirely from a routine complete blood count test. This test, a routine blood draw performed for every critically ill patient upon admission, provides all the necessary data at once for the CIPI calculation. This method, complemented by a streamlined calculation process, makes CIPI a more convenient and rapid prognostic tool compared to most indices in the MIMIC database. Moreover, our study confirms that CIPI, exhibiting significant predictive value both as a continuous variable with a linear relationship and as a categorical variable with statistically significant trend *p*-values, holds promise for guiding more personalized treatment strategies. Patients with higher CIPI scores may benefit from more aggressive interventions due to their increased risk, whereas those with lower scores might be managed with less intensive treatments, reducing unnecessary medical interventions and associated risks. However, the integration of CIPI into clinical decision-making should be approached with caution. Additional clinical trials are necessary, to further explore how CIPI’s application in clinical settings affects patient outcomes and treatment efficacy.

This study, while offering valuable insights, is subject to a few limitations. First, as a retrospective analysis, it inherently faces selection bias and potential unmeasured confounders that could influence the results. Second, due to its nature as a data mining study, the scope of data available was limited. We were unable to include comprehensive patient data such as imaging studies or genomics information as covariates in our CIPI survival analysis. This limitation may have led to an underestimation of the multifactorial nature of severe cerebral infarction prognosis, potentially affecting its predictive accuracy and generalizability to broader patient populations. Finally, the study utilized the MIMIC database, a single-center dataset with a large and diverse patient population. However, the outcomes observed may not be applicable to other clinical settings or patient populations. Therefore, multi-center studies are necessary to validate our findings and to further explore the predictive value of CIPI.

## Conclusion

This study introduces the CIPI as a robust and enhanced independent prognostic factor for critically ill patients with cerebral infarction, demonstrating linear predictability and statistically significant prognostic effectiveness both as a continuous and categorical variable. The CIPI not only matches the predictive power of established prognostic indicators but also surpasses them in simplicity and operational ease. Its derivation solely from routine complete blood count data and the potential for quick computer algorithm processing make it a practical tool for clinical use. Further multi-center studies are needed to validate these findings and to deepen the exploration of the potential utility of CIPI.

## Data availability statement

The original contributions presented in the study are included in the article/Supplementary material, further inquiries can be directed to the corresponding author.

## Ethics statement

The collection of patient information and creation of the research resource were reviewed by the Institutional Review Board at the Beth Israel Deaconess Medical Center, who granted a waiver of informed consent and approved the data sharing initiative.

## Author contributions

CS: Conceptualization, Formal analysis, Methodology, Validation, Visualization, Writing – original draft, Writing – review & editing. CZ: Funding acquisition, Supervision, Writing – original draft. GZ: Investigation, Supervision, Validation, Writing – original draft.
