# Supplementary material for: Exploring the utility of a latent variable as comprehensive inflammatory prognostic index in critically ill patients with cerebral infarction
Source: Front Neurol. 2024 Jan 15;15:1287895. doi: 10.3389/fneur.2024.1287895 (PMC10824243; doi:10.3389/fneur.2024.1287895)
Supplement: Supplementary file 1 [file Data_Sheet_1.DOCX]

Supplementary Figure


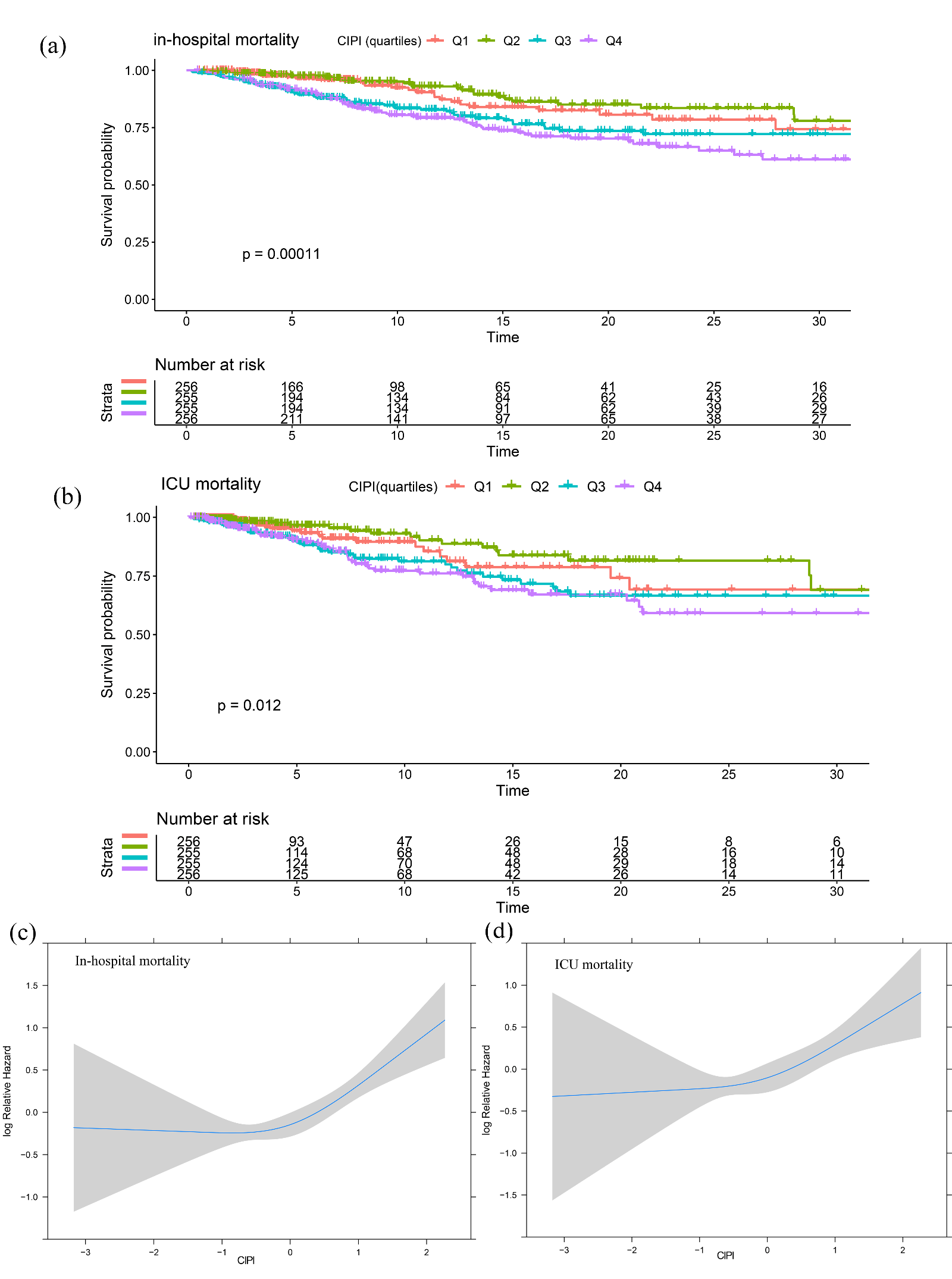


Supplementary Figure 1: Kaplan-Meier survival curves were plotted for the four groups defined by CIPI quartiles and compared using the log-rank test. a: in-hospital mortality, b: ICU mortality. We utilized the restricted cubic spline (RCS) method to investigate the linearity of the relationship between CIPI and both in-hospital mortality (c) and ICU mortality (d). By fitting the Cox proportional hazards model with a cubic spline term for CIPI, we were able to evaluate the nature of the relationship between CIPI and the mortality outcomes.


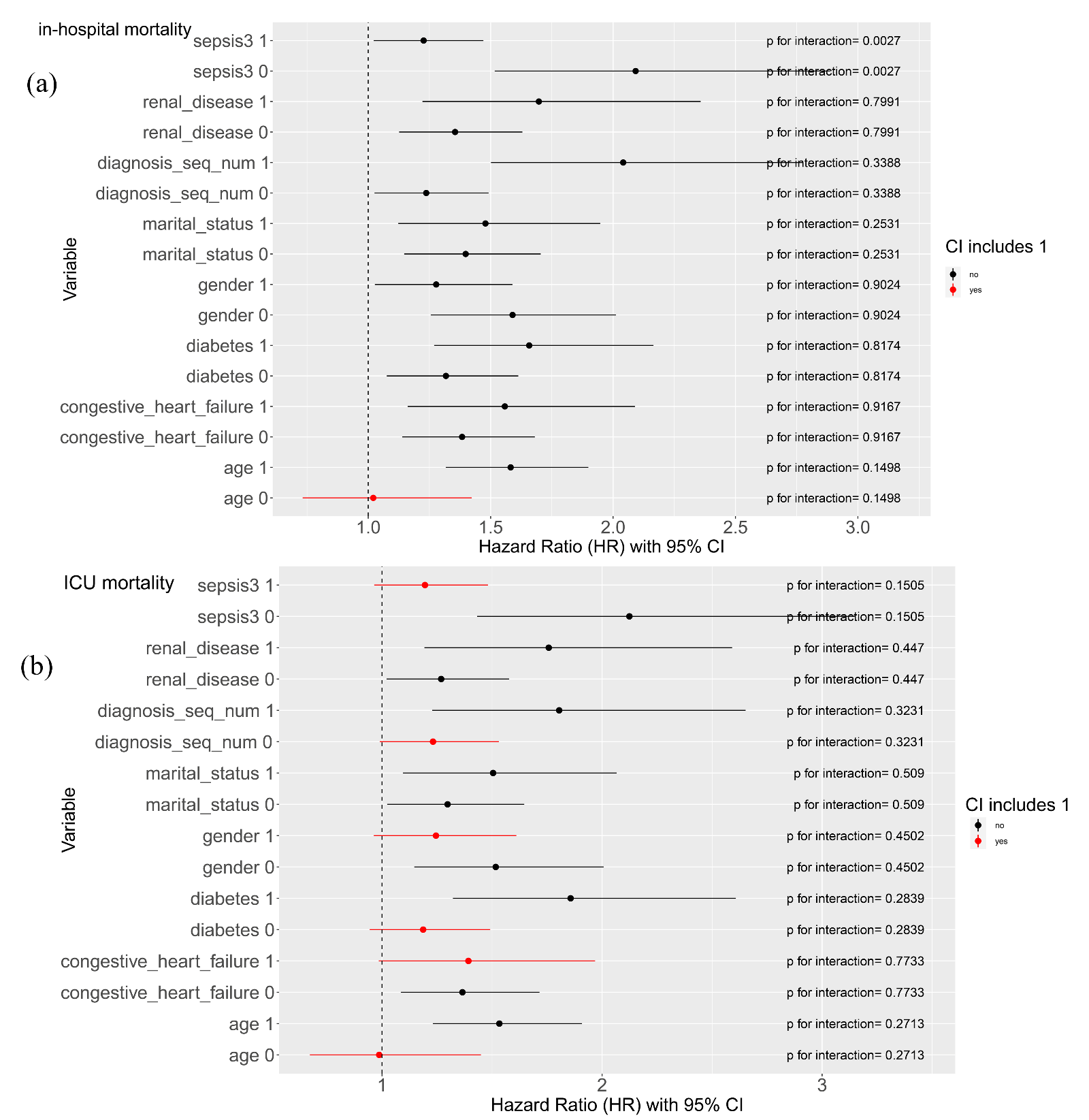


Supplementary Figure 2: We conducted subgroup and interaction analyses on CIPI using categorical variables. In the plots, the red line represents a confidence interval that includes “1”. From the graphs, we can observe that CIPI is rarely affected by interaction effects (with interaction p-values greater than 0.05), and it demonstrates statistically significant hazard ratios (HR) in the majority of the subgroups. a: in-hospital mortality, b: ICU mortality. sepsis: 1 represents the presence of severe infection, and 0 indicates no severe infection. renal_disease: 1 signifies the presence of kidney-related comorbidities, and 0 indicates the absence. diagnosis_seq_num: 1 means the patient's diagnosis of cerebral infarction is ranked first, and 0 means it is not ranked first. marital_status: 1 represents married, and 0 represents unmarried. gender: 1 denotes male, and 0 denotes female. diabetes: 1 signifies the presence of diabetes comorbidities, and 0 indicates the absence. congestive_heart_failure: 1 represents having congestive heart failure comorbidities, and 0 indicates not having them. age: 1 represents an age of 60 years or older, and 0 indicates younger than 60 years.
